# Statins and Antidepressants: A Comprehensive Review and Clinical Outlook of the Risks and Benefits of Co-prescription (2022)

**DOI:** 10.7759/cureus.32331

**Published:** 2022-12-08

**Authors:** Sai Dheeraj Gutlapalli, Dipabali Chaudhuri, Kokab Irfan Khan, Roba Al Shouli, Akhil Allakky, Asila A Ferguson, Aujala Irfan Khan, Baraa Abuzainah, Lubna Mohammed

**Affiliations:** 1 Internal Medicine Clinical Research, California Institute of Behavioral Neurosciences & Psychology, Fairfield, USA; 2 Internal Medicine, Richmond University Medical Center, Staten Island, USA; 3 Research, California Institute of Behavioral Neurosciences & Psychology, Fairfield, USA; 4 Pediatric, California Institute of Behavioral Neurosciences & Psychology, Fairfield, USA; 5 Internal Medicine, California Institute of Behavioral Neurosciences & Psychology, Fairfield, USA; 6 Psychiatry, California Institute of Behavioral Neurosciences & Psychology, Fairfield, USA; 7 General Practice, California Institute of Behavioral Neurosciences & Psychology, Fairfield, USA

**Keywords:** tricyclic antidepressants, serotonin-norepinephrine reuptake inhibitors, selective serotonin reuptake inhibitor, pharmacotherapy education, rational polypharmacy, cardiovascular disease (cvd), antidepressant drug, statin use

## Abstract

Antidepressants are the most prescribed medications in the United States, and the most frequently prescribed antidepressants are selective serotonin reuptake inhibitors (SSRIs) followed by serotonin-norepinephrine reuptake inhibitors (SNRIs), tricyclic antidepressants (TCAs), monoamine oxidase inhibitors (MAOIs), serotonin antagonist and reuptake inhibitors (SARIs), and norepinephrine-dopamine reuptake inhibitors (NDRI). On the other hand, 3-hydroxy-3-methylglutaryl coenzyme A (HMG-CoA) reductase inhibitors, also known as statins, are the most prescribed lipid-lowering medications, and because the majority of patients with cardiovascular disease (CVD) have depressive symptoms, it is essential to understand the possible drug-drug interactions these two classes of medications can have, their potential synergistic mechanisms, and possible risks. In our research, we tried to understand the facts and uncover any missing links regarding the potential risks and benefits of statins and antidepressant co-prescription in the current clinical scenario. We reviewed all the relevant information from inception up to October 2022 regarding the antidepressant and statin polypharmacy. The databases we used were PubMed and PubMed Central, and the 11 keywords were "statins," "SSRI," "SNRI," "selective serotonin reuptake inhibitors," "serotonin-norepinephrine reuptake inhibitors," "antidepressants," "HMG-CoA reductase inhibitors," "tricyclic antidepressants," "monoamine oxidase inhibitors," "serotonin antagonist and reuptake inhibitors," and "norepinephrine-dopamine reuptake inhibitors." We carefully screened each of the relevant articles, including animal and human studies. In our study, we concluded that co-prescription of statins and SSRIs/SNRIs was generally safe and should be encouraged due to the potential synergistic nature of their effects in patients with CVD and major depression, and caution is advised with all other classes of antidepressants. We would like to encourage the undertaking of large-scale observational studies and proactive postmarketing surveillance to improve our knowledge regarding this topic considering the immense clinical importance it holds by directly and indirectly affecting half the population worldwide.

## Introduction and background

Statins are 3-hydroxy-3-methylglutaryl coenzyme A (HMG-CoA) reductase inhibitors and the most commonly used lipid-lowering drug around the world [1]. We know that the most prescribed medications in the United States are antidepressants, and selective serotonin reuptake inhibitors (SSRIs) tend to be the most frequently prescribed antidepressants followed by serotonin-norepinephrine reuptake inhibitors (SNRIs) [2]. Statins in combination with SSRIs and SNRIs are usually safe, but due to the scale of the population being prescribed these medications, it is essential to delve deeper into the currently accepted facts to broaden our understanding of the interactions between these classes of medications [3].

At any one point in time, it may be estimated that more than 10% of the population of the United States may be concurrently taking an antidepressant and statin. While most of the patients are prescribed SSRI/SNRI for depressive symptoms, patients who are on statins may be concurrently taking tricyclic antidepressants (TCAs) and/or other classes of antidepressants for various other indications such as pain syndromes. Therefore, it is essential to gain a better understanding of the potential drug-drug interactions with statin and antidepressant co-prescription.

Our research focuses on the potential synergistic mechanisms between statins and antidepressants such as SSRIs and SNRIs, potential drug-drug interactions, and adverse effects. The relevant data for our review was gathered from the PubMed and PubMed Central databases. We utilized 11 keywords, which are "statins," "SNRI," "SSRI," "HMG-CoA reductase inhibitors," "selective serotonin reuptake inhibitors," "serotonin-norepinephrine reuptake inhibitors," "antidepressants," "tricyclic antidepressants," "monoamine oxidase inhibitors," "serotonin antagonist and reuptake inhibitors," and "norepinephrine-dopamine reuptake inhibitors," and the search was performed using Medical Subject Headings (MeSH) strategy. We have carefully screened and included all the relevant articles we could find since inception till October 10, 2022. All data is sourced from PubMed and PubMed Central.

## Review

A brief note on the association between cardiovascular disease and major depression

Over the next few decades, heart disease will continue to be the single biggest cause of death on the planet and the majority of patients with heart disease are known to have comorbid depression, while it is also well-established that major depression/major depressive disorder (MD/MDD) alone is an immense factor leading to long-term disability worldwide [4-6]. The risk of psychological disorders is significantly higher in patients with preexisting cardiovascular disease (CVD) than in healthy individuals, and almost 50% of patients with CVD are known to have mild-to-moderate depressive symptoms [3-5,7-9].

Furthermore, it is important to remember that metabolic syndrome (MetS) affects almost 25% of the population worldwide, and 60%-65% of the patients with mental health disorders die from causes linked to CVD, which are frequently comorbid with MetS [10].

Patients with comorbid CVD with psychological disorders have a three times lower rate of medication compliance in comparison to patients with healthy social support systems. They also have a higher risk of new-onset diabetes, malnutrition, tobacco abuse, morbid obesity, alcohol use disorder, sleep abnormalities, substance abuse, higher incidence of hospitalizations, and increased risk of all-cause mortality [4,9,11,12]. Additionally, significantly higher incidence rates of angina, myocardial infarction (MI), arrhythmias, and congestive heart failure (CHF) during initial hospital admissions are observed in CVD patients and higher rates of subsequent readmissions in patients with depression when compared to patients without depression [4,6]. A key factor in major depression is low-grade inflammation, which explains how medications that lower inflammation in CVD also ameliorate depressive symptoms [13].

On a national level, we can see that in the United States, almost nine million people are currently affected by heart failure (HF) with a 50% risk of mortality over the next five to six years, and more than four and half million of them have comorbid depression [5,9,11]. The American Heart Association also states that the prevalence of major depression is two to three times higher in patients with acute coronary syndromes (ACS) than in the general population and the incidence of ACS in patients with depression is three times higher than in patients without depression [5,6]. Every year, a million Americans are affected by ACS, with around half a million of them being previously depressed, and patients with ACS in-hospital with major depression are often reported to be depressed for a month before the cardiac event in 95% of the cases and almost 60% of them were depressed for longer than six months before the cardiac event [14]. When we look at Europe, there are currently 16 million people diagnosed with CHF and patients with major depression have a three times higher risk of developing CHF and twice the risk of mortality due to cardiac arrest compared to nondepressed patients [12]. Overall, one in five people across the planet will be depressed at some point in their lifetime [15].

Looking at the economic point of view, in the United States, more than $1 trillion has been spent over the last two decades on the medical costs related to CHF alone, with a further 30%-40% greater expenditure for patients with associated mental health disorders [6,12]. It is pertinent to mention that according to the Johns Hopkins Precursor Study, depression is a major independent risk factor for CVD, and the incidence of major depression alone by itself increased the incidence risk of CVD by up to 60% [14]. Multiple studies have proven that depression post-MI was associated with almost three times higher risk of mortality at one-year follow-up and double the risk of recurrent MI compared to patients without depression, and the highest risk of adverse outcomes was seen with treatment-resistant post-MI depression [14,15]. Various studies have also shown that hospital readmission rates are as high as 80% in one-year follow-up postcardiac events in patients with depression [16]. We need to remember that depressed patients with HF show lower motivation to follow a healthy lifestyle and have lower rates of completing cardiac rehabilitation [15,17]. Finally, the mortality from cardiac causes is known to be three times higher than from other causes in patients with depression post-MI [13].

Statins, SSRIs, and SNRIs: properties and mechanisms

We shall first discuss the basics of statin pharmacotherapy followed by SSRI and SNRI pharmacotherapy.

Statins are HMG-CoA reductase inhibitors and the most commonly prescribed medications in patients with CVD due to their effectiveness in lowering blood lipid levels [1]. Statins have cardioprotective effects due to their potent antioxidant, anti-inflammatory, and lipid-lowering properties [3,13,17]. Of note, atorvastatin also shows neuroprotective and antidepressant effects [4]. Based on affinity, statins can be divided into hydrophilic and lipophilic. Lipophilic statins are atorvastatin, simvastatin, fluvastatin, lovastatin, and pitavastatin, while pravastatin and rosuvastatin are hydrophilic statins [12]. Cholesterol and systemic inflammation are important components of neuropsychiatric disorder pathophysiology, and statins based on their mechanism of action inhibit cholesterol biosynthesis, making them effective in the treatment of dyslipidemia and CVD, which is also synergistic for reducing depressive symptoms by lowering systemic inflammation [1]. Statins, thus, protect against cardiovascular and cerebrovascular disease by lowering cholesterol and inflammation simultaneously, through direct and indirect effects on the pathophysiology of major depression, making them useful add-on medications to antidepressants in patients with CVD [16].

Statins in widely known the be safe in combination with SSRIs and have no or minimal drug-drug interactions [3]. It is a fact that the single most extensively used class of medications in the United States is antidepressants and more than 60% of these patients have been taking antidepressants for two years or longer. It is important to note that women are twice as likely to be taking an antidepressant than men, and among antidepressants, SSRIs are the most commonly prescribed medication [2].

The effects of SSRIs inhibition on platelet activity, stabilization of the vascular endothelium, and reduction in the levels of circulating inflammatory markers directly counteract effects such as higher platelet activation observed in major depression [16,18]. SSRIs are effective in curtailing sympathetic and adrenal activity hyperactivity as well as reducing stress-hemoconcentration, which are key deleterious mechanisms in CVD [19]. SSRIs are preferred in antidepressant medications in patients with CVD due to their dual action on both CVD and MDD [2,18,19]. Sertraline is considered the first-line SSRI when treating major depression post-MI [20]. SSRIs particularly sertraline reduce cardiac risk factors by reducing platelet aggregation and having positive effects on vascular endothelium in therapeutic doses along with aspirin [19]. Clinical trials have shown that SSRIs such as sertraline in combination with omega-3 supplements decreased CVD risk factors significantly [21]. SSRIs are known to reduce stress and inflammation [19]. Various studies have proven that SSRIs are efficacious in counteracting atherosclerosis, coronary artery disease (CAD), and depression [19].

SNRIs have dual serotonergic and noradrenergic activity [22]. SNRIs are the second line due to their effects on BP; SNRIs are usually well tolerated and have very few anticholinergic side effects, negligible effects on cardiac conduction and cytochrome CYP450 System in usual doses making them generally safe in patients with hepatic dysfunction [4,9,15]. The most common cardiovascular side effects of SNRIs are an increase in HR and BP [22]. There may be an increased risk of MetS due to certain SNRIs such as venlafaxine according to some reports [10]. Due to the risks of tachycardia and a rise in BP venlafaxine should be avoided in patients with HTN [23].

We should recall that the level of pro-inflammatory cytokines is predictive for the development of major depression in patients post-ACS and statins have significant attenuating effects on the inflammatory cytokines decreasing the risk of incidence for major depression [24]. Statins are known to induce tissue plasminogen activator (tPA) and inhibit plasminogen activator inhibitor 1, which is an important inhibitor of tPA [17]. This mechanism is linked to the antidepressant effect exhibited by statins [17]. Statins also have potential therapeutic implications in patients with abnormalities associated with the tPA-plasminogen pathway [17]. Multiple other anti-inflammatory drugs are known to be useful in patients with MDD, but statins are particularly useful because a majority of patients with MDD have comorbid CVD [25]. Statin therapy has been shown to reduce the incidence of depression by 32% compared to patients not prescribed statins [11]. Statins therapy is known to have positive effects on anxiety, sleep disorders, anhedonia and psychomotor retardation [8]. It is important to note that statins as add-on therapy to SSRIs have anti-depressant effects in patients with depression, but not in patients without depression [3].

Clinical trials based on patients with moderate-to-severe depression while being treated with citalopram or fluoxetine with adjuvant statin therapy showed that atorvastatin, lovastatin, and simvastatin showed improvement in depressive symptoms [6]. At a one-year follow-up after a 24-week double-blind, placebo-controlled trial of escitalopram, it was indicated that statins, especially lipophilic statins, are useful in the treatment of major depression post-ACS [15]. In a study based on multiple statins and their impact on major depression in post-CABG patients, it was inferred that simvastatin had superior antidepressant effects compared to other statins [26]. Studies have shown that concomitant use of statins and antidepressants greatly reduced the number of adverse cardiovascular events and reduced depressive symptoms in patients with severe depression [27].

The overall risk of depression was decreased by 80% in patients post-MI with statin therapy [28]. The prescriptions of antidepressants along with statins should be seriously considered to reduce the overall long-term healthcare costs, due to the synergistic nature of these medications because of depression and CVD [29]. Simvastatin is particularly useful in patients as an add-on therapy in treatment-resistant major depression [30]. Statins are also particularly useful in patients with obesity and comorbid major depression due to their effects on both depression and cholesterol levels [31]. Multiple studies have also shown that SSRIs are useful in reducing the incidence and progression of atherosclerosis when they are prescribed with statins [32].

Besides statins, various drugs such as aspirin, metformin, angiotensin-converting enzyme inhibitors, and angiotensin II receptor inhibitors have also exhibited antidepressant effects [33], whereas calcium channel blockers (CCBs), diuretics, and nitrates are associated with an increased risk of depression [34].

TCAs and statins

TCAs were available on the market since the late 1950s for the treatment of MDD [35]. TCAs structurally consist of a three-ringed structure, with an attachment of secondary or tertiary amine [35]. The secondary amines are nortriptyline, desipramine, and protriptyline, whereas the tertiary amines are amitriptyline, imipramine, clomipramine, doxepin, and trimipramine [35].

TCAs act through five different neurotransmitter pathways. The primary mechanism for their antidepressant actions is by blocking the reuptake of serotonin and norepinephrine. Similar to SSRIs and SNRIs, they act as competitive antagonists on the postsynaptic alpha cholinergic receptors, muscarinic receptors, and histaminergic receptors [35].

The TCAs that are approved for the treatment of MDD include amitriptyline, nortriptyline, protriptyline, amoxapine, doxepin, desipramine, imipramine, and trimipramine [35]. TCAs are used off-label for migraine prophylaxis, insomnia, anxiety, chronic pain, obsessive-compulsive disorder (OCD), and neuropathic pain conditions such as diabetic neuropathy and postherpetic neuralgia [35].

TCAs can cause significant adverse effects of their anticholinergic properties [35]. TCAs and their effects on the induction of the cytochrome P-450 (CYP450) system have little effect on the metabolism of statins [36]. Some studies have shown that TCAs have fewer interactions with CYP450 enzymes than SSRIs and SNRIs, but further research is needed regarding the topic [37].

The majority of antidepressant drugs are eliminated via kidneys after metabolism in the liver; hence, liver disease may lead to dangerous levels of antidepressants building up in the body [38]. Due to the lack of overlapping metabolic pathways, drug-drug interactions between TCAs and statins seem unlikely [38]. But statin metabolism could be susceptible to organic anion transporting polypeptides (OATP) inhibition by imipramine, nortriptyline, and amitriptyline, leading to an increase in drug concentration and vice versa [38].

The most important side effects of statins are an asymptomatic increase in liver transaminases and myopathy [38]. Elevations of liver enzymes are mostly transient and dose-related, usually reverting to normal values within days to weeks of continuing treatment [38]. TCAs can cause significant adverse effects of their anticholinergic properties [35]. The common adverse effects of TCAs based on effects on various neurotransmitter pathways are as follows: common cholinergic side effects include blurred vision, constipation, xerostomia, confusion, urinary retention, and tachycardia [35]. Side effects due to adrenergic blockade are orthostatic hypotension and dizziness, while histaminergic blockade may cause sedation, increased appetite, weight gain, and confusion [35].

TCAs are notoriously known for their cardiovascular adverse effects, importantly arrhythmias, due to QTc prolongation, ventricular fibrillation, and sudden cardiac death (SCD) [35]. A periodical electrocardiogram (EKG) is advisable to monitor patients on TCAs for arrhythmias due to the risk of SCD [35]. TCAs are also associated with an increased risk for seizures [35].

Acute hepatitis is rare but can be induced by various TCAS, but cross-hepatotoxicity is not commonly seen between agents [39]. TCAs are associated with a mild elevation in liver enzymes, a fact that should be observed when co-prescribing with statins [35]. TCAs are commonly avoided in patients with CAD due to their side effects, and this should be kept in mind as they may be counterproductive to statins in many patients [35]. Clomipramine has the highest rate of drug-induced liver injury among TCAs and should be avoided in patients with liver disease [35]. The fact that TCAs are commonly prescribed for off-label use makes it highly likely for a patient on statin pharmacotherapy to be concurrently placed on TCAs [35].

Serotonin antagonists and reuptake inhibitors and statins

The serotonin antagonist and reuptake inhibitors (SARIs) approved for clinical use are etoperidone, lorpiprazole, mepiprazole, nefazodone, trazodone, vilazodone, vortioxetine, niaprazine, and medifoxamine [38]. Nefazodone should not be used as the first-line antidepressant in patients treated with statins [38]. The utilization of the other SARIs may vary based on different clinical situations [38]. Nefazodone co-administration with simvastatin in healthy patients resulted in a nearly 20-fold increase in simvastatin and simvastatin acid levels [38].

There is limited availability of information regarding the interactions between SARIs and statins, even though the general mechanisms of action are nonoverlapping as in the case of most of the SARIs and statins. Further research should be conducted to gauge the risks and benefits as the prevalence of co-prescription of these two classes of medication is increasing significantly day by day.

Norepinephrine-dopamine reuptake inhibitors and statins

Bupropion is the only norepinephrine-dopamine reuptake inhibitor (NDRI) currently used in the treatment of depression, and it is a moderate inhibitor of CYP2D6 and must also be used with caution with statins [38].

Considering drugs related to P-glycoprotein (gp) transporter interactions could potentially occur with statin substrates of P-gp with antidepressants substrates or inhibitors of the transporter, resulting in an increase in drug concentrations, which needs to be studied in depth in the future [38].

Monoamine oxidase inhibitors and statins

Monoamine oxidase inhibitors (MAOIs), including nonselective MAOIs such as phenelzine, isocarboxazid, and tranylcypromine; selective type B MAO inhibitors such as selegiline; and reversible type A MAO inhibitors such as moclobemide are not prescribed as often as the other antidepressants due to apprehension regarding side effects and MAOI diet [40].

The common side effects include orthostatic hypotension, nausea, dizziness, drowsiness, and insomnia [40]. Other side effects include edema, weight gain, muscle pains, myoclonus, sexual dysfunction, and paresthesias [40]. Hepatotoxicity is a rare but important side effect of MAOIs [40].

Even in light of the lower frequency of their prescriptions, MAOIs are still utilized in the treatment of patients with atypical or treatment-resistant depression [40].

Therefore, we must be cautious of the potential risk of adverse effects due to co-prescription, especially musculoskeletal and hepatotoxic adverse effects.

Possible risks and benefits of concomitant treatment with statins and antidepressants

**Figure 1:**
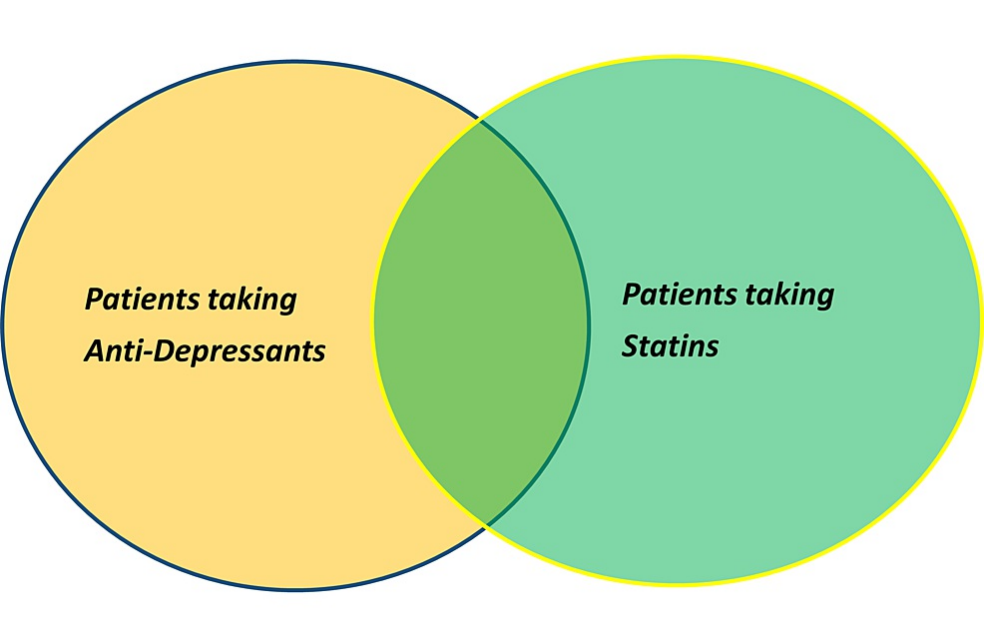
Co-prescription of statins and antidepressants. A minimum of 10% of patients are commonly prescribed both medications in the United States. It may be as high as 30%-40% in the near future. Figure credits: Sai Dheeraj Gutlapalli

We know that one-third of the patients with CAD are prescribed either SSRIs or SNRIs as antidepressant medications [41]. The response rates to antidepressants in the general population ranges currently around 50%-60%, which clearly shows a need for adjuvant therapies to current pharmacotherapy [13]. The prevalence of statin and antidepressant co-prescription is shown in Figure 1.

The occurrence of drug-drug interactions between statins and antidepressants is rare, as statins are highly selective inhibitors of HMG-CoA reductase and have no significant effects on other enzymes or direct effects on serotonin and noradrenaline receptors [42].

But it is clinically known that the antidepressant effects of statins are indirectly linked to serotonergic system modulation, which is why they act synergistically with SSRIs and SNRIs [43]. It is postulated that plasma cholesterol levels may be linked to serotonergic neurotransmission and, hence, indirectly influence antidepressant efficacy [1].

SSRIs are known to substantially improve the patient's mood post-MI and also have a relatively benign cardiac side effect profile without any significant adverse effects on heart rate (HR), blood pressure (BP), or left ventricular ejection fraction (LVEF) [16]. The most common side effects of SSRIs include xerostomia, nausea, vomiting, drowsiness, insomnia, headache, sexual dysfunction, and agitation [44]. Serious extrapyramidal symptoms such as dyskinesia, parkinsonism, akathisia, and dystonia may occur within the first 30 days of treatment [44]. There are certain important negative side effects of SSRIs that we need to remember in patients with CVD such as an increase in body weight, fasting blood glucose levels, total cholesterol, low-density lipoprotein (LDL), and triglyceride levels, but all these issues are also associated with food consumption, which may be ameliorated with a reduction in depressive symptoms, but these should be monitored in a case-by-case basis [2]. The important cardiovascular adverse effects of SSRIs, although extremely rare, include bradycardia, corrected QT interval (QTc) prolongation, orthostatic hypotension, and syncope [19]. Other rare adverse effects of SSRIs include rashes, pruritus, photosensitivity, spontaneous bruising, alopecia, and urticaria [44]. SSRIs are well tolerated in greater than 85% of patients and were observed to have significantly lower rates of cardiac events such as MI, recurrent angina, and CHF [16]. Patients with HF are known to have decreased heart rate variability (HRV), increased platelet activity, and systemic inflammation, which may be ameliorated by SSRIs [16]. Multiple clinical trials showed that SSRIs reduced morbidity and mortality in patients by almost 40% post-MI [2,19,45]. SSRIs such as sertraline are protective against weight gain and prediabetic changes in carbohydrate metabolism [2]. SSRIs are also known to reduce the risk of MI in patients affected by depression through their effects on increasing serotonin transporter affinity [46]. Among SSRIs, sertraline is known to be free of major cardiotoxic effects [19]. All SSRIs are known to inhibit CYP2D6 but do not have a significant influence on CYP3A4 [44]. Studies indicate that SSRIs did not negatively affect HR, HRV, BP, and LVEF in patients with MDD during hospitalization after acute cardiac events [13].

Numerous studies based on various antidepressants such as mirtazapine, reboxetine, citalopram, escitalopram, venlafaxine, nefazodone, paroxetine, fluoxetine, and fluvoxamine focused on the drug-drug interaction and plasma level of statins based on the effects on cytochrome system, corroborating the general safety profile of statins as add-on therapy to antidepressants [42]. Elderly patients are commonly co-prescribed antidepressants such as SSRIs and SNRIs for psychological conditions along with statins for CVD, and studies analyzing the risk of potential drug-drug interactions between SSRIs, SNRIs, and statins based on cytochrome P450 system have shown that all the statins are safe with citalopram, escitalopram, and paroxetine, while all SSRIs are safe with pravastatin, pitavastatin, and rosuvastatin [47].

Some incidents of rhabdomyolysis with pravastatin treatment in patients with MDD have been rarely reported [48]. Co-prescription of pravastatin and paroxetine may lead to a rise in mean serum glucose levels and an increase in the anticoagulation parameters such as prothrombin time, partial thromboplastin time, and international normalized ratio; these findings may be of concern in patients with increased risk of diabetes, renal disorders, and coagulation abnormalities [49]. In elderly patients, the long-term polypharmacy with antidepressants and statins may lead to an oxidation-reduction factor imbalance and high generation of reactive oxygen species, inducing rapid cellular aging [50]. It is important to note that statin use has not been linked to an increased risk of seizures, anxiety disorders, personality disorders, or suicidality [51]. The is an extremely low risk for myopathy and rhabdomyolysis with a combination of fluvoxamine with lovastatin, simvastatin, or atorvastatin [47]. Also, rare occurrences of rhabdomyolysis and transaminitis were reported in association with the concomitant use of nefazodone and simvastatin [52].

## Conclusions

Our research shows that statins and antidepressants such as SSRIs and SNRIs are generally safe in combination and can be quite useful for patients with comorbid conditions such as CVD, MDD, and MetS due to the various synergistic mechanisms of action. Lower rates of depression and higher remission rates are seen in clinical practice when patients with depressive symptoms post-cardiac events are on statins and antidepressants compared to patients prescribed monotherapy of either class of medications. Out of all the SSRIs, sertraline seems to be the safest drug of choice due to its minimal effect on cardiovascular conduction and safety profile in overdose. In general, there are very few drug-drug interactions between the two classes of medications, and regular monitoring for side effects or adverse drug reactions in patients with co-prescription of statins and antidepressants such as SSRIs and SNRIs is not necessary except in potential high-risk individuals at the discretion of the physician. TCAs, MAOIs, SARIs, and drugs such as Bupropion are also generally safe when taken along with statins even though there are rare incidences of musculoskeletal and hepatic adverse effects. In general, statins seem to be quite safe and effective when co-prescribed with all the major classes of antidepressants. We encourage further studies such as large-scale observational studies and proactive postmarketing surveillance regarding this topic as it is of immense clinical importance by directly or indirectly affecting the lives of half of the population worldwide.
